# Toxicological Parameters of a Formulation Containing Cinnamaldehyde for Use in Treatment of Oral Fungal Infections: An *In Vivo* Study

**DOI:** 10.1155/2021/2305695

**Published:** 2021-10-22

**Authors:** Danielle da Nóbrega Alves, Rafael Xavier Martins, Elba dos Santos Ferreira, Adriano Francisco Alves, Jéssica Cabral de Andrade, Tatianne Mota Batista, Josy Goldoni Lazarini, Luana Souza Amorim, Pedro Luiz Rosalen, Davi Felipe Farias, Ricardo Dias de Castro

**Affiliations:** ^1^Department of Clinical and Social Dentistry, Graduate Program in Natural and Synthetic Bioactive Products (PgPNSB), Center for Health Sciences, Federal University of Paraiba, João Pessoa PB, Brazil; ^2^Graduate Program in Molecular and Cell Biology, Center for Health Sciences, Federal University of Paraiba, João Pessoa PB, Brazil; ^3^Experimental Pharmacology and Cell Culture Laboratory, Center for Health Sciences, Federal University of Paraiba, João Pessoa PB, Brazil; ^4^Department of Physiology and Pathology, Health Sciences Center, Federal University of Paraiba, João Pessoa PB, Brazil; ^5^Graduate Program in Natural and Synthetic Bioactive Products (PgPNSB), Health Sciences Center, Federal University of Paraiba, João Pessoa PB, Brazil; ^6^Graduate Program in Dentistry (PPGO), Health Sciences Center, University of Campinas, Campinas SP, Brazil; ^7^Experimental Pharmacology and Cell Culture Laboratory, Health Sciences Center, Federal University of Paraiba, João Pessoa PB, Brazil; ^8^Department of Physiological Sciences, Center for Biological Sciences, Piracicaba Dental School, University of Campinas, Campinas, São Paula, Brazil; ^9^Laboratory for Risk Assessment of Novel Technologies (LabRisk), Department of Molecular Biology, Federal University of Paraiba, Campus I, 58051-900 João Pessoa, Brazil; ^10^Department of Clinical and Social Dentistry, Center for Health Sciences, Federal University of Paraiba, João Pessoa PB, Brazil

## Abstract

**Objective:**

We aimed to define the safety and toxicity of both isolated and embedded cinnamaldehyde using a pharmaceutical formulation for the treatment of oral fungal infections in an *in vivo* study.

**Materials and Methods:**

Acute toxicity was assessed in studies with *Galleria mellonella* larvae and *Danio rerio* embryos (zebrafish), and genotoxicity was assessed in a mouse model. The pharmaceutical formulation (orabase ointment) containing cinnamaldehyde was evaluated for verification of both *in vitro* antifungal activity and toxicity in keratinized oral rat mucosa.

**Results:**

In *Galleria mellonella* larvae, cinnamaldehyde was not toxic up to the highest dose tested (20 mg/kg) and presented no genotoxicity up to the dose of 4 mg/kg in the model using mice. However, it was found to be toxic in zebrafish embryos up to a concentration of 0.035 *μ*g/mL; LC_50_ 0.311; EC_50_ 0.097 (egg hatching delay); and 0.105 (Pericardial edema). In the orabase antifungal susceptibility test, cinnamaldehyde exhibited activity in concentrations greater than 200 *μ*g/mL. As for safety in the animal model with rats, the orabase ointment proved to be safe for use on keratinized mucosa up to the maximum concentration tested (700 *μ*g/mL).

**Conclusions:**

At the concentrations tested, cinnamaldehyde was not toxic in vertebrate and invertebrate animal models and did not exhibit genotoxic activity. In addition, when used in the form of an ointment in orabase, having already recognized antifungal activity, it was shown to be safe up to the highest concentration tested.

## 1. Introduction

Toxicity is a major challenge in pharmaceutical drug development, and many products with health applications cause undesirable side effects [1]. Factors that influence toxicity involve the chemical structure of the molecule, the dosage, and the mode of use [2]. *In vivo* studies using differing investigative animal models for assessing the toxicity of new therapeutic products are essential to support research that aims at evaluating safety and efficacy in randomized controlled clinical trials in humans [3].

The worldwide frequency of fungal infections has increased in recent years, especially in immunosuppressed individuals [4]. These infections can present superficially, but they can also bring serious systemic disease [5]. The limited number of antifungal classes [6], the increased prevalence of serious infections caused by *Candida* spp. [7], and the increase in microbial resistance to drugs [8], often due to repeated or long-term therapies [9], are motivating factors in the search for new antifungals. Compounds derived from plants have great potential to be used as antifungal agents [10]. Cinnamaldehyde, the main component obtained from the oil of the leaves and bark of *Cinnamomum zeylanicum* Blume (cinnamon), is one of the most effective inhibitors of microbial growth [11]; it presents both anti-inflammatory [12] and tissue repairing activity [13].

In a previous study, we reported on the antifungal effects of cinnamaldehyde on *Candida* species and demonstrated its strong fungicidal activity (MICs and MFCs ranging from 18.91 *μ*M to 37.83 *μ*M) [14] through a mechanism of action likely related to ergosterol complexation. In addition, fungal microcultures treated with cinnamaldehyde presented impairment of fungal cell development, with observed expression of rare pseudohyphae and the absence of chlamydeoconidio, reducing fungal biofilm by 33.75% to 64.52% (*p* < 0.0001) in low concentrations (151.3–378.3 M), in addition to being noncytotoxic to human keratinocytes and erythrocytes [15]. These results support further investigation, including evaluating toxicity and safety in animal models.

The literature has already demonstrated topical use of cinnamaldehyde in an *in vivo* model in the treatment of skin wounds resulting from *Pseudomonas aeruginosa* infection [16] and also in the form of an ointment tested in wound healing in diabetic mice [13]. However, its use as an ointment in orabase for topical treatment of fungal infections that affect the oral cavity has not yet been evaluated. The objective of this study was to define the safety and toxicity of cinnamaldehyde in in vivo models and define a pharmaceutical form which both is safe and contains cinnamaldehyde in its composition.

## 2. Materials and Methods

### 2.1. Outcome Measurements

**Figure 1:**
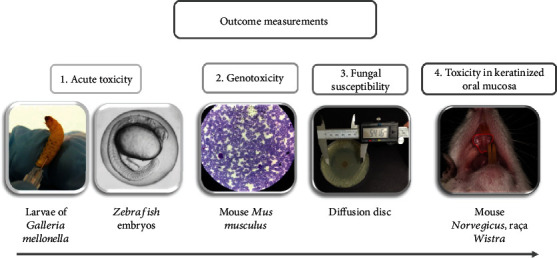
Outcome measures for cinnamaldehyde toxicology in an animal model.

The first stage of the work consisted in defining the toxicity of cinnamaldehyde in animal models with invertebrates (larvae of *Galleria mellonella*), vertebrates (zebrafish embryos *Danio rerio*), and also genotoxicity (mice). The second stage consisted of developing the orabase formulation to define its antifungal activity when tested for safety in an animal model using rats, as described in Figure 1.

### 2.2. Animals and Ethics Committee

The larvae of *G. mellonella* were kindly supplied by L. G. Leite from the Instituto Biológico, Department of Agriculture and Supply (Campinas, São Paulo, Brazil). The larvae were kept at 37°C in a BOD (Biochemical Oxygen Demand) incubator until use.

The zebrafish (*Danio rerio*) embryos were provided by the zebrafish facility of the Department of Molecular Biology, Federal University of Paraiba (João Pessoa, Brazil). Zebrafish adults (wild type strain) were kept at 26 ± 1°C under a 14 : 10 h (light : dark) photoperiod. The water quality was maintained by activated carbon filtration, conductivity at 750 ± 50 *μ*S, and dissolved oxygen above 95% saturation. The fish were fed daily with commercial food (Tetra ColorBits, Sarandi, Brazil) and *Artemia* sp. nauplii and were also monitored for abnormal behavior or disease development. To obtain embryos, an egg trap was placed overnight in a tank containing male and female specimens (2 : 1 ratio) one day prior to testing. One hour after the beginning of the light cycle, eggs were collected with a Pasteur pipette and rinsed with E3 medium (5 mM NaCl, 0.17 mM KCl, 0.33 mM CaCl_2_, and 0.33 mM MgSO_4_) for subsequent selection of embryos using a stereomicroscope (80x magnification). Viable fertilized eggs were selected for embryo toxicity assays (LISBON 2020).

For the genotoxicity tests, male Swiss albino mice (Mus musculus), at five to six weeks of age, weighing approximately 30 g were used. Provided by the Animal Production Unit of the Institute for Research in Drugs and Medicines (IPeFarM), at the Federal University of Paraiba, heterogeneous male rats of the species Rattus norvegicus, Wistar breed, and weighing an average of 300 g were used to evaluate toxicity in keratinized oral mucosa.

All study conditions were approved by the Ethics Commission in Animal Use in Research (CEUA) of the Federal University of Paraiba, Approval No. 2342111119.

### 2.3. Acute Toxicity Tests Using *Galleria mellonella* Larvae

In order to define whether the toxicity profile of cinnamaldehyde is dose dependent in relation to the survival of *Galleria mellonella* larvae, the hypothesis that the substance is not toxic in this invertebrate animal model was tested. Nine doses of cinnamaldehyde (0.15, 0.31, 0.62, 1.25, 2.5, 5.0, 10.0, and 20.0 mg/kg) were tested on larvae weighing 200 to 300 mg without signs of melanization and selected randomly for each group (*n* = 15/group). The cinnamaldehyde (test group) and the 0.9% NaCl (control group) were injected (10 *μ*L) into the hemocele of each larva through the last left proleg using a 25 *μ*L Hamilton syringe (Hamilton, Reno, NV). The vehicle (dimethylsulfoxide) used to dilute the substance was also tested for its lethality. All groups were incubated at 30°C, and larvae survival was monitored at selected intervals for up to 72 h. Larvae with no movement to the touch were considered dead [17, 18].

### 2.4. Acute Toxicity Test Using Zebrafish Embryos

The Fish Embryo Acute Toxicity (FET) test was conducted with cinnamaldehyde according to OECD's guideline number 236 (OECD, 2013). Zebrafish embryos with up to 3 hpf (hours postfertilization) of age were exposed to five crescent concentrations of cinnamaldehyde (0.56, 0.28, 0.14, 0.07, 0.035, and 0.017 *μ*g/L). For each concentration tested, a 24-well plate was prepared containing 20 fertilized eggs (1 embryo per well) exposed to the test sample and 4 embryos were exposed only to E3 medium (internal controls). An additional plate containing embryos exposed to the E3 medium was also assayed. The exposure was performed for 96 h, and the embryos were analyzed every 24 h for the apical endpoints: egg coagulation, lack of somite formation, lack of detachment of the tail-bud from the yolk sac, and lack of heartbeat. Additionally, sublethal effects were also recorded daily, being the following: eye malformation, otolith malformation, mouth malformation, spine malformation, body pigmentation, egg hatching delay, developmental delay, yolk sac edema, body malformation, pericardial edema, head edema, blood clotting, and undersize. The number of deaths (lethal endpoints) and sublethal effects were used to calculate the LC_50_ (median lethal concentration) and EC_50_ (median effective concentration), respectively, by *probit* regression analysis [19].

The exposures were under static conditions (without renovation of test sample or E3 medium). Observations were performed in a stereomicroscope (×80 magnification) and photographed (Zeiss). After 96 h, surviving larvae were euthanized with eugenol and properly discarded.

### 2.5. Genotoxicity

To evaluate the genotoxicity of the sample, the micronucleus assay was performed using the peripheral blood of the test mice. Groups of five male (*n* = 5/group) albino Swiss mice (Mus musculus), aged five to six weeks, weighing approximately 30 g, received by gavage a dose of 1200 *μ*g/mL (4 mg/kg). One negative control group (0.9% NaCl solution and 2% Tween 80) and a positive control group (cyclophosphamide, 50 mg/kg) were included. At 48 hours of treatment, the animals were submitted to a small incision in the tail to obtain a blood sample (10 *μ*L) for making blood extensions. After drying, the slides were stained with panotic staining (Panótico Rápido®) for further analysis under an optical microscope. For each animal, three blood extensions were prepared and a minimum of 2000 erythrocytes counted per animal to determine the number of micronucleated erythrocytes. After *in vivo* genotoxicity tests, the animals were anesthetized with a solution of xylazine (16 mg/kg i.p.) and ketamine (100 mg/kg i.p.) and then euthanized by cervical dislocation [20]. The experimental procedure was approved by the Ethics Committee on the Use of Animals of the Federal University of Paraíba under Protocol No. 2342111119.

### 2.6. Fungal Susceptibility Test

For this analysis, a standard strain of *Candida albicans* of the American Type Culture Collection (ATCC) 90028 was used. Agar diffusion testing was performed using the well drilling technique. The fungal inoculum was adjusted and standardized to a concentration of 2.0 × 10^6^ CFU · mL^−1^. Cinnamaldehyde was tested in the form of an ointment in orabase (and is also being analyzed for patent filing at the National Institute of Industrial Property-INPI, under number BR 10 2019 025510 2). The tested concentrations were 12.5, 25, 50, 100, 200, 300, 400, 500, 600, 700, and 800 *μ*g/mL.

The Petri dishes containing agar were prepared homogeneously (all groups). Each plate received a total of 24 mL of Sabouraud Dextrose agar (SDA, São José do Pinhais, PR, Brazil). With the aid of an inoculation loop, fungal colonies of the strain were collected, suspended in 5 mL of Sabouraud Dextrose Broth (SDB) and placed in a greenhouse at 35°C. After 24 h, the inoculum was adjusted, and a sterile swab was introduced for inoculation in the form of streaks on the agar surface in three directions. The previously inoculated plates had small portions of the solid culture medium removed to form the wells, using the upper edge of a test tube. As such, roughly 90 mg of orabase ointment (containing cinnamaldehyde) was placed inside a central hole made in the agar. After incubation, the diameters of the fungal development inhibition halos were measured with a caliper. The tests were carried out in triplicate, and miconazole gel (20 mg/g) (Janssen-Cilag Farmacêutica Ltda., São Paulo, SP, Brazil) was used as a positive control [21].

### 2.7. Toxicity Study on Keratinized Rat Oral Mucosa

Fifty heterogeneous male Wistar rats of the species Rattus norvegicus, weighing on average 300 g, were obtained from the Animal Production Unit of the Institute for Research on Drugs and Medicines (IPeFarM), of the Federal University of Paraíba, and randomly divided into 10 groups (5 rats/group) to administer different concentrations of an antifungal ointment (25 *μ*g/mL, 50 *μ*g/mL, 100 *μ*g/mL, 200 *μ*g/mL, 300 *μ*g/mL, 400 *μ*g/mL, 500 *μ*g/mL, 600 *μ*g/mL, and 700 *μ*g/mL) and a negative control (ointment without the active ingredient). Random numbers were generated using the “*random between*” function in Microsoft Excel to place the animals in their respective groups. The animals were kept in plastic cages and kept in an environmentally controlled room (temperature 24°C, relative humidity at 60%, and a light/dark cycle of 12 hours) with free access to fresh solid pellet food and water.

Three applications were made at 8-hour intervals (113 mg of ointment, on average) for 15 days. The animals were carefully handled and monitored to avoid possible risks of accidents, poisoning, and/or contamination. The application was also performed very delicately so that it did not hurt the rat's palatal mucosa. To avoid possible toxicity in the rats, concentrations were only increased if the test group did not experience any unwanted reactions, such as abrupt weight loss, inflammation, and/or mucosal ulceration. That is, if any concentration in the orabase ointment containing cinnamaldehyde caused undesirable effects, the subsequent higher concentration would not be tested. In the event of any suffering being untreatable, the animal would be subjected to immediate euthanasia.

After the ointment application period, all animals were submitted to histological analysis based on an excisional biopsy of the hard palate mucosa. The tissues obtained were fixed in 10% buffered formaldehyde (pH 7.4) for 72 hours. The histological sections, 4 *μ*m thick, were placed on glass slides and stained using the hematoxylin and eosin technique for further evaluation of histological events occurring in each group using light microscopy. All procedures from the beginning of the study until the moment of euthanasia were performed to avoid suffering and reduce any discomfort or pain in the animals [22].

Classification of histological events considers the presence of inflammatory cells, changes in connective tissue cell populations, characteristics of the amorphous intercellular substance, and fibrous intercellular substance. The material was classified by a single examiner, and the histological events observed in the mucosa region for each experimental group were described after treatment.

### 2.8. Statistical Analysis

The results are expressed as the mean ± standard deviation. Statistical analysis of the data was performed using analysis of variance (ANOVA), followed by the Tukey test (*p* < 0.05). For the cytotoxicity of zebrafish embryos, the mean lethal concentration values (LC_50_) and the drug concentration values that induce half the maximum effect (EC_50_) were calculated by probit regression analysis.

## 3. Results and Discussion

### 3.1. Toxicity against *Galleria mellonella* Larvae

**Figure 2:**
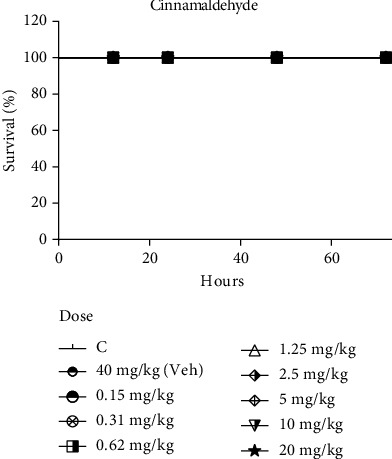
Systemic toxicity of cinnamaldehyde in the Galleria mellonella larvae model. The larvae received treatments with control (C; 0.9% NaCl solution) and vehicle (DMSO) at a dose of 40 mg/kg and cinnamaldehyde at doses of 0.15, 0.31, 0.62, 1.25, 2.5, 5.0, 10.0, and 20.0 mg/kg for 72 hours. All groups were compared with the control group (C). *p* > 0.05, log-rank test.

The acute systemic toxicity of cinnamaldehyde was assessed using the alternative model with *G. mellonella* larvae. High doses of this substance were injected into the larvae, and their survival was monitored for a period of 72 h. As seen in Figure 2, cinnamaldehyde presented no toxic effects on the larvae when administered in concentrations with recognized *Candida* spp. antibiofilm activity (10 × Minimum Inhibitory Concentration − 37.8 *μ*M (378 *μ*M)) [15]. This concentration corresponds to a dose of 20 mg/kg. It was not possible to find the lethal dose capable of killing 50% of the larvae. This represents important information when assessing the toxicity of cinnamaldehyde in an invertebrate animal model and suggests performing tests in animals with greater physiological complexity [23].

We are the first to study the acute toxicity of cinnamaldehyde using *G. mellonella* larvae. This model, in addition to being viable, low-cost, and validated [17, 24], provides answers quickly, as the larvae have a short life cycle (approximately 6 weeks), and there is no need for specialized equipment. Additionally, the immune system of this invertebrate animal is similar to that of mammals [25, 26].

In a study using *G. mellonella*, the authors assessed toxicities of fruit extracts. The extracts of *Sageretia elegans* and *Byrsonima arthropoda* were shown to cause acute toxicity effects in the larvae, and *Spondias mombin* reduced the survival of the larval population by almost 50% as compared to the vehicle (*p* < 0.05) [27]. Another study evaluated the acute toxicity of *Eugenia brasiliensis* fruit pulp extract and found that the extract had no toxic effects on these same larvae when administered in antibiofilm concentrations (10 × MIC) [23]. There are no toxicity studies on cinnamaldehyde or extracts of plants that contain cinnamaldehyde, against *G. mellonella* larvae. Our results revealed that none of the systemic doses (0.15–20 mg/kg) resulted in larval death. This difference may be explained due to the differing parts of the plant (peel and fruit pulp extract) used in the studies. Cinnamaldehyde is extracted from the skin of species of the genus Cinnamomum, and in the other studies mentioned above, the compounds under analysis were extracted from fruit pulps. Further, the values of the doses tested differed, being calculated based on the antibiofilm value of each substance.

### 3.2. Toxicity in Zebrafish Embryos

In order to determine whether cinnamaldehyde was toxic to zebrafish development, its embryos were exposed to increasing concentrations of the substance. This is the first report characterizing the effects of cinnamaldehyde on the development of zebrafish.

**Figure 3:**
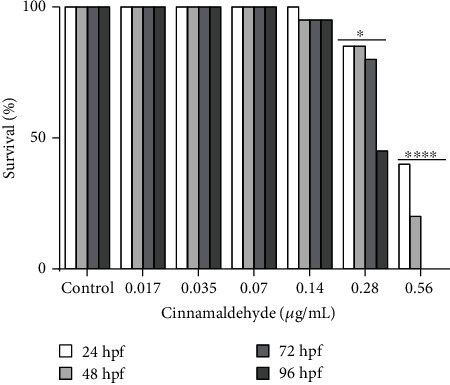
Zebrafish survival after exposure to cinnamaldehyde. Effect of cinnamaldehyde on zebrafish embryo and larvae survival rate at 96 h of exposition. The presence of any lethal effect was counted as mortality. *N* = 20 embryos/group. hpf: hours postfertilization. *p* = 0.024 at a concentration of 0.28 *μ*g/mL and *p* < 0.0001 at a concentration of 0.56 *μ*g/mL when compared with the control.

The survival rate and malformations in zebrafish were measured, and cinnamaldehyde presented dose-dependent effects on embryo survival up to 96 hpf (Figure 3). Survival, as presented in the graph for the concentrations of 0.017, 0.035, 0.07, and 0.14 *μ*g/mL, did not differ significantly from the negative control, but at the highest concentrations tested, the embryo's survival decreased by 26.25% (for the concentration of 0.28 *μ*g/mL vs. the control; *p* = 0.024) and by 85% (for the concentration of 0.56 *μ*g/mL vs. the control; *p* < 0.0001).

**Figure 4:**
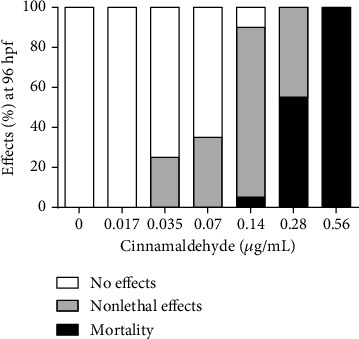
General overview of the cumulative effects of cinnamaldehyde on zebrafish embryos and larvae at 96 h of exposure. No effect: morphological characteristics similar to the nontreated organisms; nonlethal effects: presence of sublethal changes; mortality: presence of mortality endpoints. *N* = 20 embryos/group. hpf: hours postfertilization.

The embryotoxicity of cinnamaldehyde included mortality endpoints and malformations.  Figure 4 presents an overview of the cumulative effects of cinnamaldehyde on zebrafish embryos and larvae at 96 h of exposure. The number of nonlethal and lethal outcomes worsened as concentrations increased.

Table 1 presents a general panel of cinnamaldehyde lethality in relation to the zebrafish development parameters. In the morphological analysis, it was observed that cinnamaldehyde induced changes in zebrafish embryos starting at a concentration of 0.035 *μ*g/mL (LOAEL), e.g., delays in egg hatching (EC_50_ = 0.097 *μ*g/mL) and pericardial edema (EC_50_ = 0.105 *μ*g/mL). The lowest concentration tested for the absence of adverse effects on the embryos (NOAEL) was 0.017 *μ*g/mL.

This vertebrate animal model has been shown to be useful for toxicology, especially in drug screening and human disease studies [28]; it is a reliable, controllable, and reproducible model [29]. Yet, some authors suggest that for assessing toxicity, this experimental model is very sensitive, with the initial stages of development being commonly the most sensitive to chemical exposure [30].

**Figure 5:**
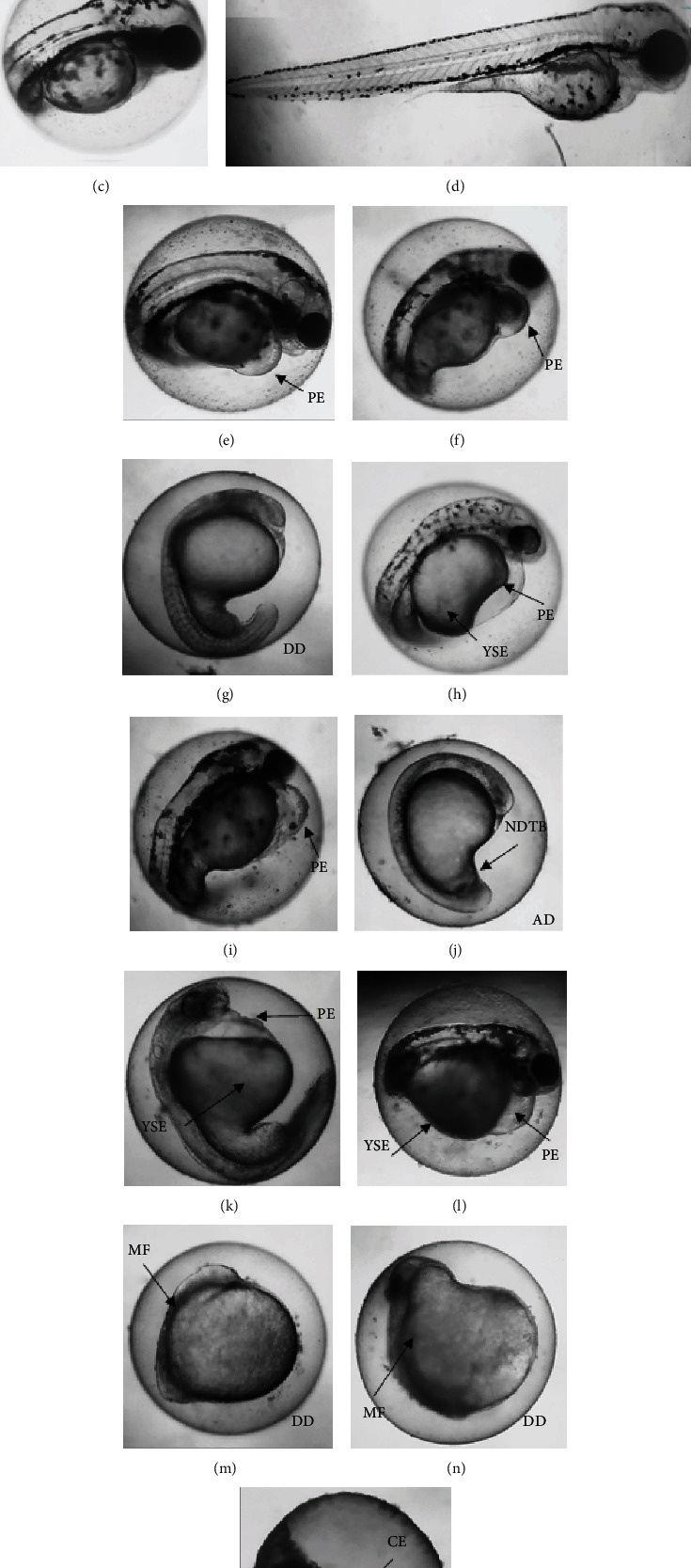
Lethal and nonlethal effects observed in zebrafish embryos and larvae after exposure to increasing concentrations of cinnamaldehyde during 96 h. (a–d) control organisms with normal development after 24, 48, 72, and 96 hpf, respectively, exposed only to the E3 medium; (e, f) embryos with 96 hpf, exposed to 0.07 and 0.035 *μ*g/mL, respectively, both with pericardial edema (PE); (g) embryo with 24 hpf exposed to 0.14 *μ*g/mL showing developmental delay (DD); (h, i) embryos with 48 and 96 hpf, respectively, exposed to 0.14 *μ*g/mL, both with pericardial edema (PE); (j) 24 hpf embryo, exposed to 0.28 *μ*g/mL, showing developmental delay (DD) and nondetachment of the tail base (NDTB); (k, l) embryos with 48 and 96 hpf exposed to 28 *μ*g/mL presenting pericardial edema (PE) and yolk sac edema (YSE), respectively, and (l) yolk sac edema (YSE); (m, n) embryos with 24 and 48 hpf, respectively, exposed to 56 *μ*g/mL of cinnamaldehyde, presenting malformation (MF) and developmental delay (DD); (o) coagulated embryo (CE) after 72 h exposure to 56 *μ*g/mL.

Often, the toxic responses of substances are conserved beyond the zebrafish and are thus also found in toxicological studies in humans [31]. Yet, in species such as rats, mice, and guinea pigs, studies have already shown that acute cinnamaldehyde toxicity is relatively low, with LD_50_ values (50% lethal dose) ranging from 0.6 to more than 2 g/kg [32]. In this study, it was shown that cinnamaldehyde induced mortality at the two highest concentrations tested; on the other hand, the toxic concentrations caused morphological changes such as egg hatching delay, yolk sac edema, body malformation, pericardial edema, and developmental delay (Figure 5). This may be due to differing embryo polarities, which cause the molecule to act by affinity (lipophilicity), a chemical characteristic of the molecule under analysis [33].

The high mortality found at the desired concentrations may be due to small variations in the rate of embryo development, such as the variation in the number of embryos emerging from their chorions at the time of treatment (malformations occur more frequently while the embryos are in the chorion), the ability of oil components to cross the embryonic barrier [34], accumulation of blood close to the anterior portion of the yolk sac, weakening of the vessels, pericardial edema, necrotic tissue effects which interrupt the structure of the heart, or increased pressure resulting from the edema [35].

### 3.3. Evaluation of Genotoxicity

A 4 mg/kg single dose of the treatment sample in the animals did not induce an increase in the number of micronucleated erythrocytes in peripheral blood (7.8 ± 0.3), as compared to the control group (8.6 ± 0.4). As expected, cyclophosphamide induced a significant increase in the number of micronucleated erythrocytes (18.4 ± 0.5; *p* < 0.05), as to the control group (8.6 ± 0.4) (Table 2).

Taking these results into account, it can be inferred that cinnamaldehyde at a dose of 4 mg/kg does not present genotoxic effects on the erythroid system. However, a previous study has shown that cinnamaldehyde at doses of 850, 1700, and 2550 mg/kg is capable of inducing micronucleus formation in liver cells [36]. The genotoxicity of cinnamaldehyde has also been demonstrated in the literature in an *in vitro* model using a reversal assay. The authors proved that cinnamaldehyde presents no mutagenic activity in the genotoxicity test, since it did not interact with DNA to transmit and trigger mutations [37].

### 3.4. Fungal Susceptibility Test

In order to evaluate the therapeutic efficacy of the orabase ointment containing cinnamaldehyde, its antifungal activity was estimated. This pharmaceutical form was chosen due to its advantages in providing easier application, in spreading the product over the affected region, and for promoting adhesion at the tissue lesion site. The formulation also comes flavored, which improves product acceptance and increases patient adherence, all while acting as a mucoprotective barrier for ulcerated oral lesions [38].

*In vitro* and *in vivo* test agreement is important, since to date, in both models, and in the concentrations and doses respectively tested, cinnamaldehyde presented antifungal activity without cytotoxic effects [15] and in the invertebrate model presented toxicity with no genotoxic effects as well. Thus, the results obtained in these tests help to estimate and define orabase ointment product concentrations that may be used in further studies on safety.

**Figure 6:**
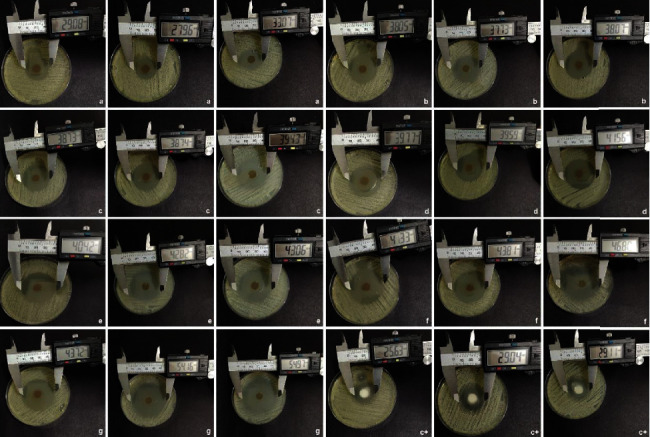
Images of ointment inhibition halos. ^∗^By concentration: (a) 200 *μ*g/mL; (b) 300 *μ*g/mL; (c) 400 *μ*g/mL; (d) 500 *μ*g/mL; (e) 600 *μ*g/mL; (f) 700 *μ*g/mL; (g) 800 *μ*g/mL; C+(positive control-miconazole).

**Figure 7:**
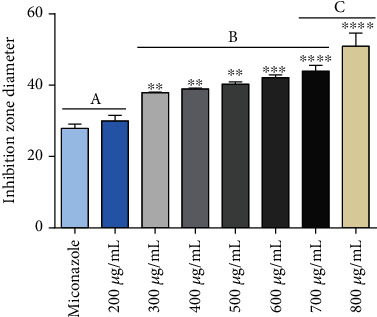
Antifungal effects of the cinnamaldehyde test ointment against the tested *Candida albicans* strain. The graph represents the mean ± SD of three independent experiments carried out in triplicate. One-way ANOVA followed by Tukey's posttest was performed for comparison between groups; ^∗∗∗∗^*p* < 0.0001, ^∗∗∗^*p* < 0.001, ^∗∗^*p* < 0.01, and ^∗^*p* < 0.05 in relation to the positive control.

For good test resolution, agar thickness and uniformity are determinants [21]. Thus, the Petri dishes used in the assay for antifungal activity were prepared in a standardized way with the same agar volume and a central hole of the same diameter. The strain was exposed to different concentrations of cinnamaldehyde (12.5, 25, 50, 100, 200, 300, 400, 500, 600, 700, and 800 *μ*g/mL), and antifungal activity was estimated using the diameter of the inhibition zone. It was observed that the halo formed by the ointments was concentration dependent, with no statistical difference between miconazole and the ointment containing cinnamaldehyde at 200 *μ*g/mL. Statistical differences from the positive control started at 300 *μ*g/mL, yet there was no difference between the 300 and 700 *μ*g/mL concentrations (*p* > 0.05). Ointments at concentrations of between 12.5 and 100 *μ*g/mL did not present antifungal activity (data not shown). According to our results, the orabase ointments containing cinnamaldehyde inhibited *Candida albicans* growth, a species commonly present in the oral cavity of human beings [5]. The results are presented in Figures 6 and 7.

The literature had already demonstrated the *in vitro* effects of cinnamaldehyde (isolated) against species of the genus *Candida* [15]. The effect of *Cinnamomum zeylanicum* Blume essential oil, a product that contains a significant amount of cinnamaldehyde in its composition, has also been demonstrated in the form of a spray for treatment of oral candidiasis associated with the use of removable prosthesis in a phase II clinical trial [39]. However, the effect of cinnamaldehyde in the form of an ointment in orabase for use in the oral cavity has not yet been defined. Thus, in addition to antifungal activity, it was necessary to test the safety of this new pharmaceutical form in animals to arrive at phase I clinical trials in humans.

### 3.5. Toxicity Study: Rat Keratinized Oral Mucosa

**Figure 8:**
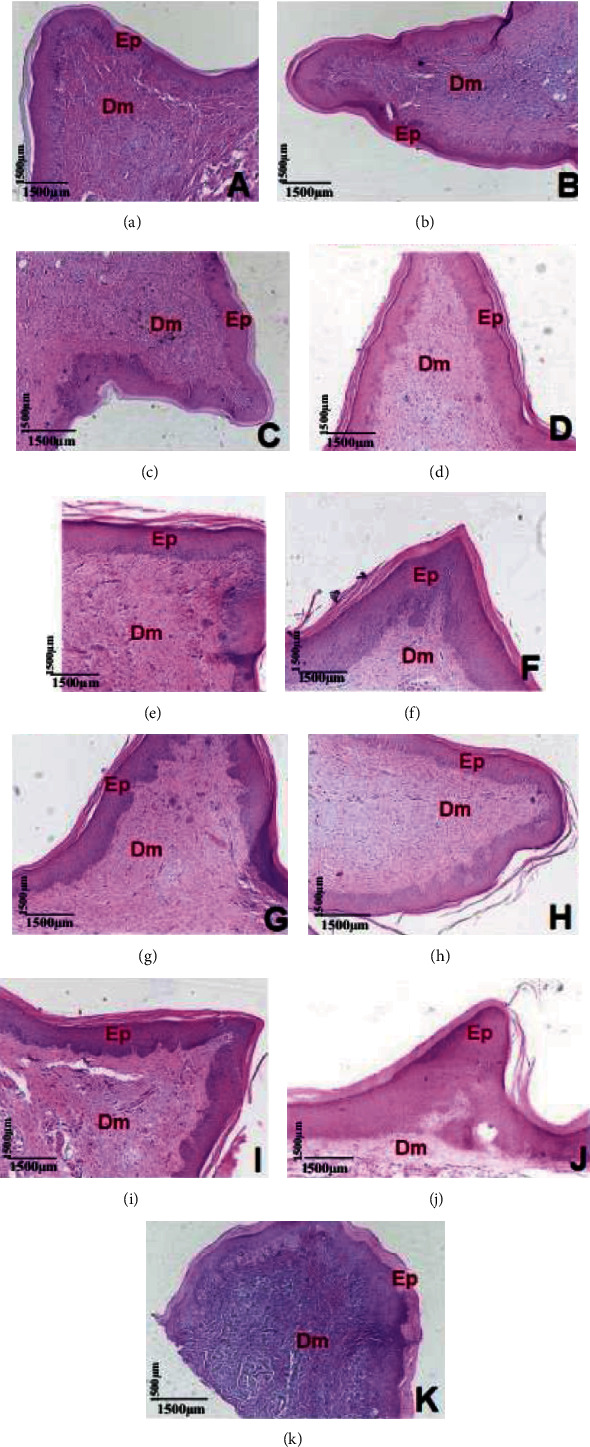
Test rat hard palate histology after orabase ointment application containing cinnamaldehyde in different concentrations (H/E, ×10): (a) 25 *μ*g/mL; (b) 50 *μ*g/mL; (c) 100 *μ*g/mL; (d) 200 *μ*g/mL; (e) 300 *μ*g/mL; (f) 400 *μ*g/mL; (g) 500 *μ*g/mL; (h) 600 *μ*g/mL; (i) 700 *μ*g/mL; (j, k) control ointment using base alone.

The results revealed that at all concentrations tested (25, 50, 100, 200, 300, 400, 500, 600, and 700 *μ*g/mL), the animals remained healthy, not presenting clinical and/or macroscopic evidence of inflammation. Histological analyses of palate specimens presented preservation of epidermis (Ep), keratinocytes, and basal cells without dermatopathological changes and preservation of the dermis (Dm) which presented no vascular and/or cellular changes, being compatible with normal mucosa at all doses tested. The results are presented in Figure 8.

These results were expected since the literature had already demonstrated the anti-inflammatory capacity of cinnamaldehyde [11]. The activity involves induction of apoptosis; inhibition of cell proliferation in immune responses mediated by monocytes/macrophages; suppression of nitric oxide production, with positive regulation of costimulatory molecules (CD80 and CD69) and standard recognition receptors (toll-like receptor 2 (TLR2), complement receptor (CR3) [40]); and lower levels of TNF-*α* and IL-6 [12].

This anti-inflammatory and regenerative capacity was also tested in an *in vivo* model. Certain authors have tested cinnamaldehyde in topical form, demonstrating its ability to help heal wounds infected with *Pseudomonas aeruginosa*. This is because cinnamaldehyde induces lower levels of interleukin-17 (IL-17), vascular endothelial growth factor (VEGF), and nitric oxide [16]. Another study evaluating the effectiveness of an ointment prepared from hydroethanolic extract of *Cinnamomum verum*, containing 11.26% cinnamaldehyde in its composition, revealed an acceleration in wound healing in diabetic mice. This occurs due to a greater proliferation of fibroblasts, collagen deposition, reepithelialization, and keratin biosynthesis [13]. Tissue regeneration has also found to be favored through induction of peripheral vasodilation through activation of transient receptor potential of ankyrin-1 (TRPA1) in mice [41].

## 4. Conclusions

Cinnamaldehyde was shown to be nontoxic in vertebrate and invertebrate animal models, while also presenting no genotoxic activity. When used in the form of an ointment in *orabase*, with recognized antifungal activity against *Candida albicans*, cinnamaldehyde presented no clinical or histological evidence of inflammatory effect in animal mucosa.

## Figures and Tables

**Table 1:** Effects of cinnamaldehyde on developmental parameters of zebrafish early stages after 96 h of exposure.

96 h: embryotoxicological adverse effects	LC_50_	EC_50_	LOAEL	NOAEL
*Mortality (all lethal effects)*	0.311	NA	0.14	0.07
Coagulation of viable embryos			0.14	0.07
Lack of somite formation			—	—
Lack of heartbeat			0.28	0.14
Nondetachment of the tail bud from the yolk sac			0.28	0.14
*Nonlethal effects*				
Eye malformation	—	—	—	—
Otolith malformation	—	—	—	—
Mouth malformation	—	—	—	—
Spine malformation	—	—	—	—
Body pigmentation	—	—	—	—
Egg hatching delay	—	0.097	0.035	0.017
Yolk sac edema	—	NC	0.035	0.017
Body malformation	—	NC	0.14	0.070
Pericardial edema	—	0.105	0.035	0.017
Head edema	—	—	—	—
Blood clotting	—	—	—	—
Undersize	—	—	—	—
Developmental delay	—	NC	0.14	0.070

LOAEL: lowest observed adverse effect level in *μ*g/mL; NOAEL: no observed adverse effect level in *μ*g/mL; —: not observed; LC_50_: median lethal concentration in *μ*g/mL; EC_50_: median effective concentration values in *μ*g/mL; NA: not applicable; NC: not calculated.

**Table 2:** Effect of single-dose cinnamaldehyde (4 mg/kg) and cyclophosphamide (50 mg/kg) administrations on the number of micronucleated mice erythrocytes, in peripheral blood after 48 hours of treatment.

Groups	Dose (mg/kg)	No. of micronucleated cells
Control	—	8.6 ± 0.4
Cyclophosphamide	50	18.4 ± 0.5^a^
Cinnamaldehyde	4	7.8 ± 0.3

Data presented as mean ± standard error of the mean of five animals. ^a^*p* < 0.05 compared to the control group (12% Tween 80), analyzed by ANOVA followed by Dunnet and Tukey.

## Data Availability

All data used to support the findings of this study may be released upon application to the corresponding author (Ricardo Castro), who can be contacted at rcastro@ccs.ufpb.br.
